# Emergence of *tet(X4)* and *bla*_NDM−5_ in clinical isolates of *Salmonella* Typhimurium 1,4,[5],12:i:- from Jinhua, China: epidemiological and genomic characterization

**DOI:** 10.3389/fmicb.2026.1744620

**Published:** 2026-03-24

**Authors:** Chen Yang, Shuying Zhu, Zhifeng Pang, Bin Wu, Junliang Wu, Jingchao Shi

**Affiliations:** 1 Jinhua Center for Disease Control and Prevention, Jinhua, China; 2 Department of Clinical Laboratory, Affiliated Jinhua Hospital, Zhejiang University School of Medicine (Jinhua Municipal Central Hospital), Jinhua, China

**Keywords:** *bla*_NDM−5_, genomic epidemiology, multidrug resistance (MDR), plasmid mobilization, *Salmonella* Typhimurium, *tet(X4)*

## Abstract

**Background:**

The monophasic variant of *Salmonella enterica* serovar Typhimurium (1,4,[5],12:i:-) has emerged as a major multidrug-resistant (MDR) clone worldwide. This study aimed to characterize the genomic epidemiology and the occurrence of critical antimicrobial resistance genes in *S*. 1,4,[5],12:i:- ST34 isolates from diarrheal patients in Jinhua, China.

**Methods:**

A total of 51 clinical isolates were collected from diarrheal patients in Jinhua, Zhejiang Province, China, between 2022 and 2024. Antimicrobial susceptibility testing was performed according to CLSI 2023 guidelines, and whole-genome sequencing was used to determine sequence types, resistance genes, and plasmid structures.

**Results:**

Most isolates carried a conserved MDR backbone including *bla*_TEM − 1B_*, sul2, tet(B), floR*, and *aac(6')-Iaa*, conferring resistance to ampicillin, tetracycline, chloramphenicol, and trimethoprim-sulfamethoxazole, while all remained susceptible to polymyxin B. Phylogenomic analysis confirmed all belonged to ST34, showing diversification of accessory resistomes over time. Two isolates harbored last-line resistance determinants: *tet(X4)* on a mosaic IncHI1/FIA plasmid and *bla*_NDM−5_ on an IncHI2 plasmid with markedly increased conjugation efficiency at 26 °C, suggesting environmental facilitation of plasmid transfer.

**Conclusions:**

This study demonstrates the persistence of a stable MDR core within regional *S*. 1,4,[5],12:i:- ST34 populations and the sporadic emergence of high-risk plasmids carrying *tet(X4)* and *bla*_NDM−5_. These findings underscore the urgent need for integrated One Health surveillance to limit further dissemination of clinically important resistance in *Salmonella*.

## 1 Introduction

*Salmonella enterica* remains a leading cause of foodborne illness worldwide, imposing substantial morbidity and mortality each year. Among its >2,600 serovars, *Salmonella* Typhimurium is one of the most prevalent in human and animal infections, including in China where long-term surveillance has documented its stable predominance among non-typhoidal *Salmonella* (NTS) serovars (Wang et al., 2022).

In recent decades, a monophasic variant of *S*. Typhimurium (antigenic formula 1,4,[5],12:i:-) has emerged and disseminated globally (Hendriksen et al., 2011). This variant, characterized by the loss of phase 2 flagellar antigen expression while retaining close genetic and phenotypic similarity to biphasic *S*. Typhimurium, is now recognized as a high-priority zoonotic pathogen (Kim et al., 2015). Multiple molecular approaches—including Multilocus Sequence Typing (MLST), and whole-genome phylogenetics—have confirmed its close evolutionary relatedness to classical *S*. Typhimurium.

Three major epidemic lineages of monophasic *S*. Typhimurium—the Spanish, American, and European clones—have been described. Among them, the European clone dominated by sequence type 34 (ST34) has achieved global dissemination and is now considered the most successful pandemic lineage (Sun et al., 2020). ST34 is typically associated with the Ampicillin-Streptomycin-Sulfonamide-Tetracycline (ASSuT) resistance profile and frequently carries genomic islands or mobile elements conferring tolerance to heavy metals such as copper and mercury (Wu et al., 2025; Tassinari et al., 2020). More recently, ST34 isolates worldwide have accumulated additional resistance determinants against critically important antimicrobials, including extended-spectrum cephalosporins, fluoroquinolones (García et al., 2014).

In China, genomic surveillance studies indicate that ST34 has become one of the dominant NTS lineages across human, animal, and food sources (Wang et al., 2023; Yang et al., 2015). Large-scale genomic datasets further demonstrate that antimicrobial resistance in *Salmonella* is shaped by both clonal expansion and horizontal gene transfer on a global scale (Wang et al., 2025).

Of particular concern is the emergence of last-line resistance genes such as *bla*_NDM−5_ and *tet(X4)*.

While *tet(X4)* has been detected in *Salmonella* from different serovars and countries, and some studies have included phenotypic confirmation and conjugation assays (Wang et al., 2021; Zhang et al., 2025), many reports were based on retrospective or surveillance datasets with limited accompanying clinical and epidemiological metadata (Wang et al., 2023, 2025). Our study complements these works by providing prospectively collected clinical isolates with integrated phenotypic, genomic, and plasmid-level analyses. Similarly, carbapenemase-producing *Salmonella* carrying *bla*_NDM−5_ are increasingly reported but remain uncommon compared to other Enterobacterales (Sun et al., 2025; Zeng et al., 2023; Zhou et al., 2025).

To better characterize the local epidemiology and resistance features of monophasic *Salmonella* Typhimurium, we investigated the molecular characteristics, antimicrobial resistance phenotypes, and genomic epidemiology of *S*. 1,4,[5],12:i:- isolates collected from diarrheal patients in Jinhua, Zhejiang Province, China, between 2022 and 2024.

This study complements existing large-scale genomic surveys by incorporating prospectively collected clinical isolates with linked phenotypic data, high-resolution plasmid characterization including complete circular assemblies, and experimental assessment of conjugation dynamics under environmentally relevant temperatures. Notably, two isolates were found to carry the last-line resistance genes *tet(X4)* and *bla*_NDM−5_, respectively, each located on self-transmissible plasmids.

By integrating antimicrobial susceptibility testing, whole-genome sequencing, and functional conjugation assays, this study provides regionally grounded evidence on the occurrence and transmission potential of last-line resistance determinants in *S*. 1,4,[5],12:i:-. These data contribute to a more nuanced understanding of how high-risk resistance genes circulate at the human–food–environment interface and support the need for integrated One Health surveillance.

## 2 Materials and methods

Fifty five monophasic *S*. Typhimurium (antigenic formula 1,4,[5],12:i:-) isolates were collected from sentinel hospitals across Jinhua City, Zhejiang Province, China, between January 2022 and December 2024. The monitoring network comprised 11 sentinel hospitals located in 9 districts/counties within Jinhua, including one specialized children's hospital and ten general hospitals. The studies involving human participants were approved by the Medical Ethics Review Committee of Jinhua Center for Disease Control and Prevention (Approval Number: 202306) and the Ethics Committee of Jinhua Central Hospital (Approval No. 2026-Research Ethics-45).

### 2.1 Inclusion criteria

Patient population: isolates were obtained from outpatients or inpatients presenting with diarrhea as the primary symptom, with a documented history of suspected food exposure within 3–7 days prior to symptom onset.

Sample type: clinical specimens consisted of fresh stool or rectal swabs collected during routine diagnostic workup.

Sampling period: a total of 11 isolates were collected in 2022, 17 in 2023, and 23 in 2024, ensuring temporal coverage of three consecutive years.

Sample quality: only non-duplicate isolates were included, defined as one isolate per patient per episode of infection.

### 2.2 Exclusion criteria

Non-target serovars: isolates identified as other *Salmonella* serovars were excluded.

Incomplete clinical data: strains lacking essential patient or epidemiological information (e.g., exposure history, clinical presentation) were excluded to maintain data integrity.

### 2.3 Identification and confirmation

Initial strain identification was performed using Matrix-Assisted Laser Desorption/Ionization–Time of Flight Mass Spectrometry (MALDI-TOF MS; Bruker Daltonics, Germany) with a score threshold >2.0 for reliable species identification. Serovar determination was subsequently confirmed using *Salmonella* diagnostic antisera (SSI Diagnostica, Denmark) following the Kauffmann–White scheme. Quality control strains included *Escherichia coli* ATCC 25922, *Salmonella* Typhimurium ATCC 14028, and *Pseudomonas aeruginosa* ATCC 27853 to ensure the accuracy and reproducibility of identification procedures.

### 2.4 Antibiotic susceptibility testing

Antimicrobial susceptibility testing of all *Salmonella* 1,4,[5],12:i:- isolates was performed using the broth microdilution method with in-house–prepared 96-well microdilution panels, following the guidelines of the Clinical and Laboratory Standards Institute (CLSI, 2023; M100, 33rd edition).

A total of fifteen antimicrobial agents representing major therapeutic classes were tested: gentamicin, ciprofloxacin, nalidixic acid, ampicillin, ampicillin/sulbactam, cefotaxime, ceftazidime, cefoxitin, cefazolin, imipenem, trimethoprim-sulfamethoxazole, azithromycin, tetracycline, chloramphenicol, and polymyxin B.

Antibiotic powders were purchased from Shanghai Yuanye Bio-Technology Co., Ltd. (Shanghai, China) and dissolved in sterile water or appropriate solvents according to CLSI recommendations. Two-fold serial dilutions were prepared in cation-adjusted Mueller–Hinton broth (CAMHB) to achieve final concentration ranges of 0.25–512 mg/L, depending on the antimicrobial agent.

Each isolate was tested in triplicate. The inoculum was adjusted to 5 × 10^5^ CFU/ml and incubated at 35 ± 1 °C for 16–20 h. Minimum inhibitory concentrations (MICs) were determined visually as the lowest concentration with no visible growth. *Escherichia coli* ATCC 25922 and *Pseudomonas aeruginosa* ATCC 27853 were used as quality control strains in every batch.

Interpretation of results followed CLSI 2023 clinical breakpoints, and isolates were categorized as susceptible (S), intermediate (I), or resistant (R). Multidrug resistance (MDR) was defined as resistance to three or more antimicrobial classes. For polymyxin B, susceptibility was determined using the CLSI M100 standard broth microdilution method. Because CLSI does not currently provide interpretive criteria for polymyxin B against Enterobacterales, FDA breakpoints were applied (susceptible ≤ 2 mg/L; resistant ≥4 mg/L).

### 2.5 Conjugation assay and transfer frequency determination

Liquid mating experiments were conducted to evaluate the horizontal transferability of plasmids carrying *tet(X4)* and *bla*_NDM−5_. The donor strains consisted of Gram-negative isolate ZJJH22SAL03 [*tet(X4)*] and isolate ZJJH23SAL27 (*bla*_NDM−5_), while sodium azide-resistant *Escherichia coli* J53 served as the recipient.

Donor and recipient strains were streaked onto Mueller–Hinton agar plates and incubated at 37 °C for 18 h. Single colonies of each strain were inoculated into 3 ml Luria-Bertani (LB) broth and grown overnight at 37 °C with shaking for the recipient and without shaking for the donor. Each overnight culture (500 μl) was transferred into 4.5 ml fresh LB broth and incubated for 4 h under the same conditions. Equal volumes (500 μl each) of donor and recipient cultures were combined in 4 ml LB broth and incubated statically at 26 °C and 37 °C overnight.

The mating mixtures were serially diluted (10^0^, 10^−1^, 10^−2^, 10^−3^) and plated on selective LB agar containing sodium azide (100 mg/L) supplemented with either meropenem (2 mg/L, for *bla*_NDM−5_) or tigecycline [2 mg/L, for *tet(X4)*]. Plates were incubated at 37 °C for 18–20 h. Putative transconjugants were subcultured onto the same selective media to confirm purity, followed by antimicrobial susceptibility testing and Polymerase Chain Reaction (PCR) amplification to verify the presence of the respective resistance genes (Yang et al., 2022; Wang et al., 2021; Jiang et al., 2024).

PCR confirmation of *tet(X4)* and *bla*_NDM−5_ was performed using previously published primers: for *tet(X4)*, F: 5′-CTGATTCGTGTGACATCATCTTTTG-3′ and R: 5′-GTTAAATTCCCATTGGTCAGATTA-3′; for *bla*_NDM−5_, F: 5′-GGTTTGGCGATCTGGTTTTC-3′ and R: 5′-CGGAATGGCTCATCACGATC-3′. PCR conditions consisted of initial denaturation at 95 °C for 5 min, followed by 30 cycles of 95 °C for 30 s, 52 °C for 30 s, and 72 °C for 45 s, with a final extension at 72 °C for 5 min. Amplified products were confirmed by agarose gel electrophoresis (204 bp for *tet(X4)* and 621 bp for *bla*_NDM−5_) (Ji et al., 2020; Vodianyk et al., 2025).

Conjugation frequency was calculated as the ratio of transconjugant CFU to donor CFU.

### 2.6 Whole-genome sequencing and bioinformatics analysis

Whole-genome sequencing was performed on all 51 *S*. 1,4,[5],12:i:- isolates using the Illumina NovaSeq 6000 platform (paired-end, 150 bp reads). In addition, hybrid sequencing was conducted for two representative isolates—ZJJH22SAL03 [harboring *tet(X4)*] and ZJJH23SAL27 (harboring *bla*_NDM−5_)—combining Oxford Nanopore long reads with Illumina short reads to obtain complete and circularized assemblies of chromosomes and plasmids.

Raw Illumina reads were quality-trimmed using fastp v0.23.2, and hybrid assemblies for ZJJH22SAL03 and ZJJH23SAL27 were generated with Unicycler v0.5.0 (Wick et al., 2017). Short-read assemblies for the remaining isolates were produced using SPAdes v3.15.5 (Bankevich et al., 2012). Genome annotation was performed with Prokka v1.14.6 (Seemann, 2014), and assembly completeness was assessed using BUSCO v5.4.4 (Simão et al., 2015). Antimicrobial resistance genes were identified via the ResFinder 4.3 (FloRensa et al., 2022) and CARD databases (Alcock et al., 2020), applying thresholds of: ≥90% nucleotide identity; ≥80% gene coverage. while plasmid replicon types were determined using PlasmidFinder v2.1 (Carattoli et al., 2014). Insertion sequences and mobile genetic elements (MGEs) were annotated with ISfinder (Siguier et al., 2006) (accessed July 2025).

Core-genome SNPs were identified using Snippy v4.6.0 by mapping reads to *S*. Typhimurium SL1344 (NC_016810.1), with minimum coverage 10x and minimum variant allele fraction 0.9. A SNP distance matrix was generated and used to construct Neighbor-Joining phylogenies via BacWGSTdb v2.0 (http://bacdb.cn/BacWGSTdb). Trees were visualized and annotated using iTOL v6. (https://itol.embl.de). For contextual analysis, complete genomes of *S*. 1,4,[5],12:i:- carrying *tet(X4)* or *bla*_NDM−5_ were retrieved from NCBI RefSeq (accessed July 2025) and analyzed separately.

To enhance reproducibility, example commands, parameter settings, and analysis workflows are publicly available at (https://github.com/shijc088-ui/ST34-Jinhua-2024-).

### 2.7 Genetic context and fitness determinant analysis

The genetic surroundings of *bla*_NDM−5_ and *tet(X4)* in isolates ZJJH23SAL27 and ZJJH22SAL03, respectively, were examined to investigate their structural organization and potential mobilization mechanisms. Flanking regions of each resistance gene were extracted from the assembled plasmid sequences and compared against reference plasmids available in the NCBI nucleotide database using BLASTn with 95% identity, 80% coverage, and E-value 1e-5 thresholds. Synteny visualization was performed using Easyfig v2.2.3, and circular plasmid maps generated using Proksee (https://proksee.ca/). To explore potential dissemination determinants, additional genomic features were investigated: prophage detection. Prophage regions were identified using PHASTER (http://phaster.ca/). Only intact prophages with completeness score 90 were retained for analysis; questionable and incomplete prophages were excluded. Presence of *sopE* was assessed by tBLASTn using the SOPE protein (UniProt O52623) with thresholds of 90% identity, 80% query coverage, and E-value 1e-10. Integrons were detected using IntegronFinder v2.0 via Galaxy. Only integrons carrying the intI1 integrase gene were classified as class 1 integrons. Heavy-metal resistance determinants were identified using BacAnt, applying ≥90% identity and ≥80% coverage thresholds. Detailed workflows and example commands are provided in the GitHub repository.

## 3 Results

### 3.1 Overview of the epidemiological investigation

From 2022 to 2024, *S*. 1,4,[5],12:i:- isolates were obtained through routine surveillance in Jinhua, Zhejiang Province, China, encompassing 11 sentinel hospitals across nine administrative divisions, including one pediatric specialty hospital and 10 general hospitals. All isolates were recovered from stool or rectal swabs of patients presenting with acute diarrhea and a suspected history of foodborne exposure. A total of 51 isolates were identified during this 3-year period, including 11 in 2022, 17 in 2023, and 23 in 2024.

Among the confirmed cases, 28 (54.9%) were male and 23 (45.1%) female (male-to-female ratio = 1.22:1). Patient ages ranged from 6 months to 93 years, with the highest prevalence in the 0–3 years group (20/51, 39.2%), followed by those aged 55–93 years (16/51, 31.4%), 4–20 years (8/51, 15.7%), and 21–55 years (7/51, 13.7%). A distinct seasonal trend was observed, with 3 cases (5.9%) during January–March, 18 cases (35.3%) during April–June, and 30 cases (58.8%) during July–September, peaking in the third quarter. Geographically, cases were distributed across seven of the nine divisions, with the highest numbers from Yongkang City (22 cases), followed by Yiwu City (7 cases), Wucheng City (7 cases), Pujiang County (6 cases), Lanxi City (3 cases), Panan City (1 case) and Dongyang City (5 cases).

### 3.2 Strain identification and drug sensitivity results

All isolates were identified as *Salmonella enterica* using the Bruker MALDI-TOF MS system (Bruker Daltonics, Germany). Subsequent serotyping with commercial antisera (SSI, Denmark) according to the Kauffmann–White scheme confirmed the antigenic profile 1,4,[5],12:i:-, consistent with the monophasic variant of *S*. Typhimurium.

High resistance rates (>50%) were observed for ampicillin (94.1%, 48/51), tetracycline (90.2%, 46/51), chloramphenicol (60.8%, 31/51), and trimethoprim-sulfamethoxazole (54.9%, 28/51). Resistance to gentamicin (7.8%, 4/51), ciprofloxacin (9.8%, 5/51), nalidixic acid (2.0%, 1/51), ampicillin-sulbactam (13.7%, 7/51), ceftazidime (7.8%, 4/51), imipenem (2.0%, 1/51), and azithromycin (5.9%, 3/51) remained below 15%. Moderate resistance was observed for cefotaxime (21.6%, 11/51) and cefazolin (21.6%, 11/51). All isolates were susceptible to polymyxin B. 35 isolates (68.6%) were classified as MDR (Table 1).

**Table 1:** Antimicrobial resistance of 51 S. 1,4,[5],12:i:- isolates (2022–2024).

**Antibiotic class**	**Antibiotic**	**2022 (*n* = 11)**	**2023 (*n* = 17)**	**2024 (*n* = 23)**	**Total (*n* = 51)**	***P-value (χ^2^)***
Aminoglycosides	Gentamicin (GEN)	1 (9.1%)	1 (5.9%)	2 (8.7%)	4 (7.8%)	0.934
Quinolones	Ciprofloxacin (CIP)	0 (0.0%)	1 (5.9%)	4 (17.4%)	5 (9.8%)	0.224
	Nalidixic acid (NAL)	0 (0.0%)	0 (0.0%)	1 (4.3%)	1 (2.0%)	0.537
β-lactams	Ampicillin (AMP)	11 (100.0%)	14 (82.4%)	23 (100.0%)	48 (94.1%)	0.041
Ampicillin/Sulbactam (AMS)	4 (36.4%)	1 (5.9%)	2 (8.7%)	7 (13.7%)	0.047
Cephalosporins	Cefotaxime (CTX)	3 (27.3%)	4 (23.5%)	4 (17.4%)	11 (21.6%)	0.784
	Ceftazidime (CAZ)	0 (0.0%)	3 (17.6%)	1 (4.3%)	4 (7.8%)	0.166
	Cefazolin (CFZ)	3 (27.3%)	4 (23.5%)	4 (17.4%)	11 (21.6%)	0.784
Carbapenems	Imipenem (IMP)	0 (0.0%)	1 (5.9%)	0 (0.0%)	1 (2.0%)	0.361
Folate inhibitors	Trimethoprim/Sulfamethoxazole (SXT)	6 (54.5%)	10 (58.8%)	12 (52.2%)	28 (54.9%)	0.916
Macrolides	Azithromycin (AZM)	1 (9.1%)	2 (11.8%)	0 (0.0%)	3 (5.9%)	0.259
Tetracyclines	Tetracycline (TET)	11 (100.0%)	15 (88.2%)	20 (87.0%)	46 (90.2%)	0.462
Phenicols	Chloramphenicol (CHL)	8 (72.7%)	10 (58.8%)	13 (56.5%)	31 (60.8%)	0.650
Polymyxins	Polymyxin B (PB)	0 (0.0%)	0 (0.0%)	0 (0.0%)	0 (0.0%)	—

Resistance rates (%) were calculated based on the number of resistant isolates out of the total tested per year.

Antibiotics were classified according to their drug class. Statistical comparisons among years were performed using the Chi-square (χ^2^) test, and P-values > 0.05 indicate no significant difference in resistance rates over the study period, whereas P < 0.05 indicates a significant difference.

GEN, gentamicin; CIP, ciprofloxacin; NAL, nalidixic acid; AMP, ampicillin; AMS, ampicillin-sulbactam; CTX, cefotaxime; CAZ, ceftazidime; CFZ, cefazolin; IMP, imipenem; SXT, trimethoprim-sulfamethoxazole; AZM, azithromycin; TET, tetracycline; CHL, chloramphenicol; CT, polymyxin B.

### 3.3 Phylogenetic analysis and comparative resistance gene analysis

Genomic characterization demonstrated that all *S*. 1,4,[5],12:i:- isolates belonged to the ST34 lineage, a globally prevalent clonal group strongly associated with multidrug resistance. Within this lineage, the isolates were distributed across several core genome sequence types (cgSTs), including cgST-16838, cgST-17296, cgST-18410, cgST-17521, and cgST-9106.

**Figure 1:**
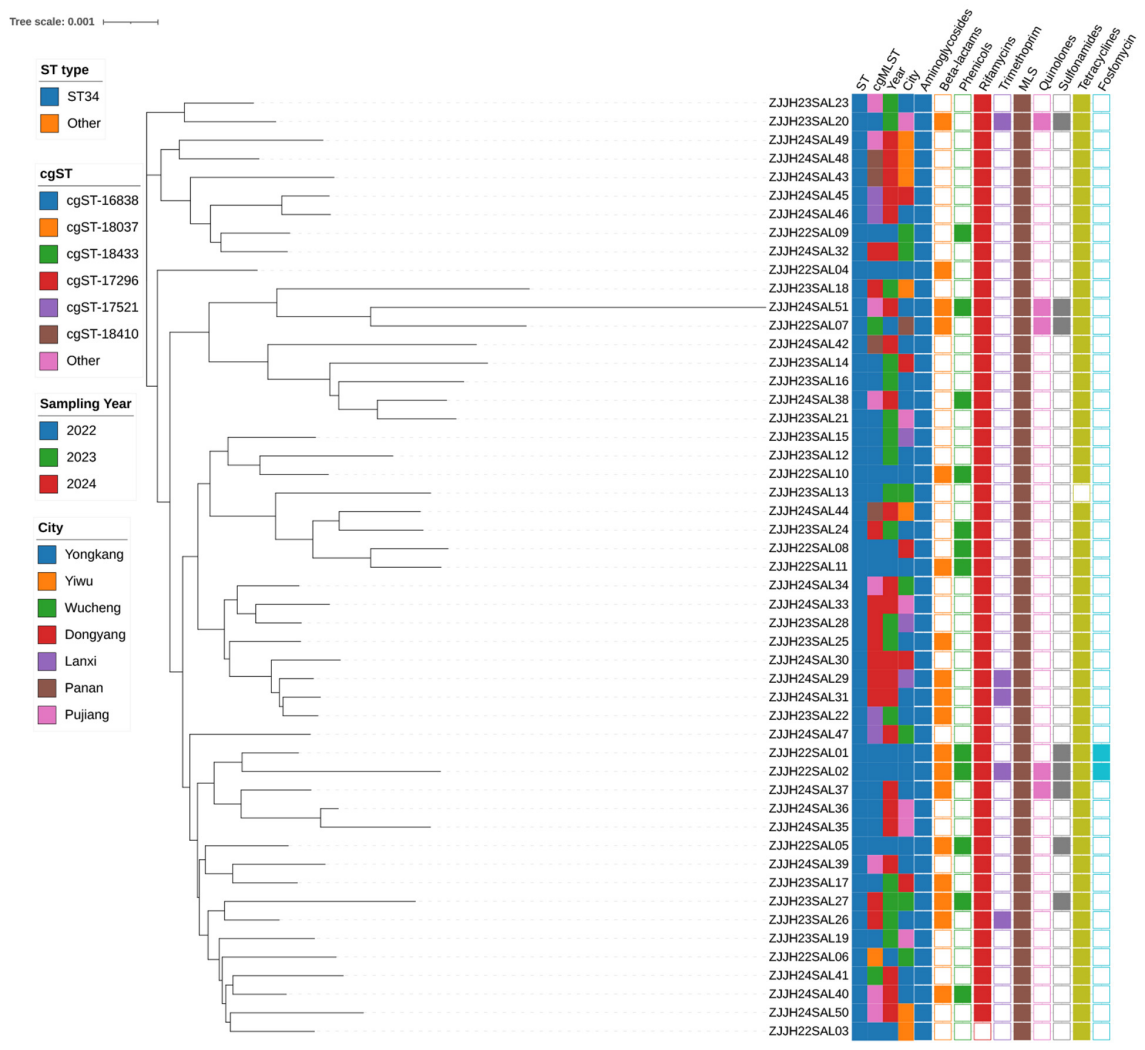
Phylogenetic analysis of *tet(X4)*-positive *S*. 1,4,[5],12:i:- isolates from NCBI and ZJJH22SAL03. Core-genome phylogeny of *tet(X4)*-carrying *S*. 1,4,[5],12:i:- isolates, including strain ZJJH22SAL03 (cgST-16838) and publicly available genomes from NCBI. ZJJH22SAL03 clusters tightly with GCA_049312515.1 and several Chinese isolates, while a separate cgST-17296 clade and a divergent UK genome (branch length ~0.010) indicate at least two independent acquisitions of *tet(X4)* within the ST34 lineage. These findings suggest parallel introductions of *tet(X4)* and highlight the evolutionary plasticity of ST34 *Salmonella* under antimicrobial selection.

The majority of isolates shared a conserved set of resistance determinants that form the backbone of the ST34 resistome. These included *aac(6')-Iaa* and *aph(6)-Id* (aminoglycoside resistance), *bla*_TEM − 1B_ (β-lactam resistance), *sul2* (sulfonamide resistance), *tet(B)* (tetracycline resistance), and *floR* (phenicol resistance). This gene constellation is consistent with the internationally recognized MDR profile of ST34. One isolate, ZJJH22SAL03, deviation from this core resistance pattern by carrying the *tet(X4)* gene. This gene encodes a flavin-dependent monooxygenase capable of inactivating tigecycline, one of the last-resort agents for complicated infections caused by MDR Enterobacterales. The same isolate also carried *lnu(G)* (conferring lincosamide resistance), *qnrS1* (a plasmid-mediated quinolone resistance determinant), and *bla*_TEM − 1B_, collectively broadening its resistance spectrum and highlighting its elevated clinical risk. Additionally, another isolate, ZJJH23SAL27, was found to harbor *bla*_NDM−5_, a carbapenemase gene conferring resistance to nearly all β-lactams, including carbapenems (Figure 1).

### 3.4 ZJJH22SAL03 carrying *tet(X4)*—High-resolution comparative genomics and phylogeny

**Figure 2:**
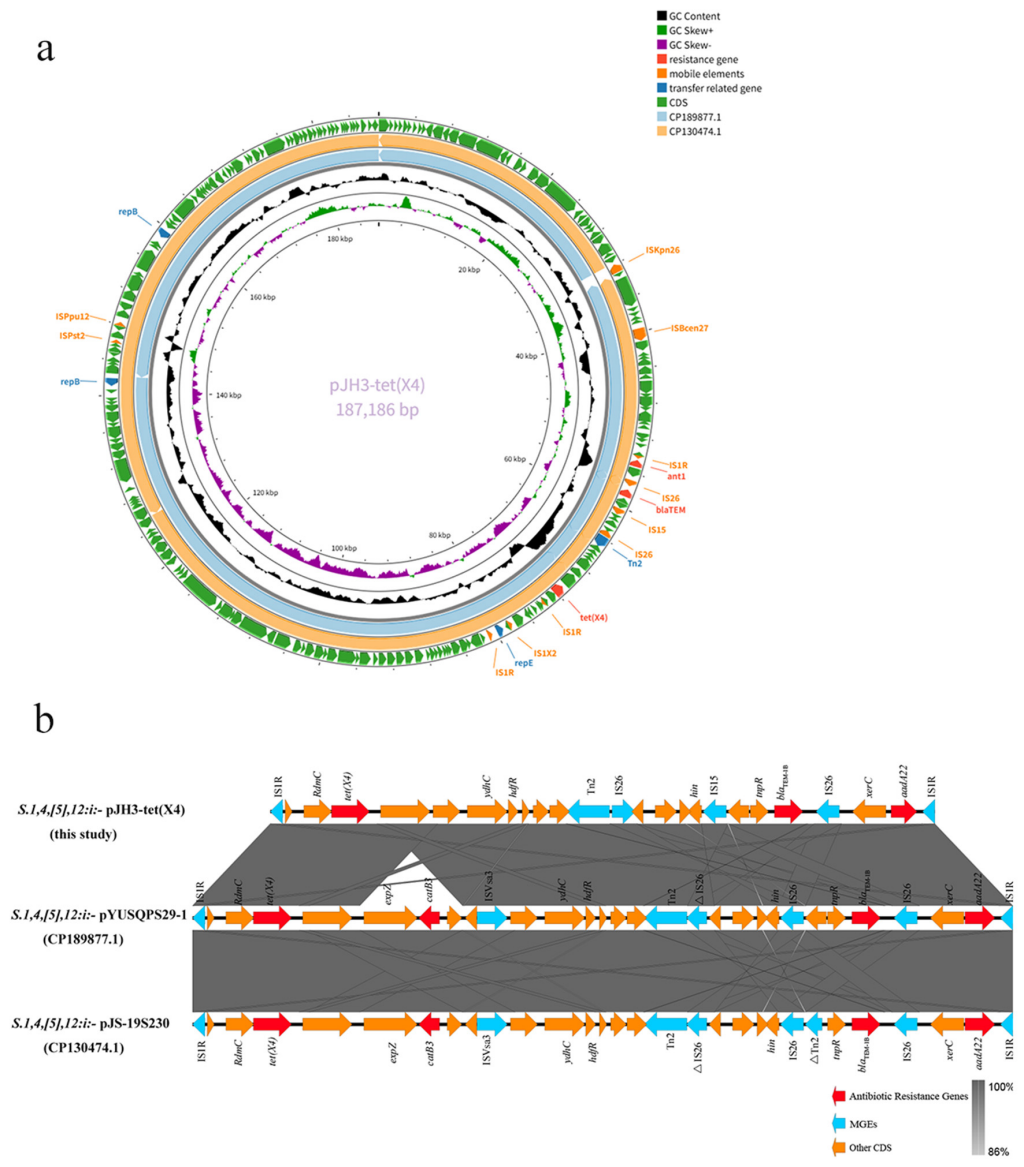
**(a)** Circular map of the *tet(X4)*-bearing plasmid pJH3-*tet(X4)* from *S*. 1,4,[5],12:i:- isolate ZJJH22SAL03.Circular representation of pJH3-*tet(X4)* [187,186 bp, IncHI1/FIA (HI1)/HI1B (R27)]; **(b)** Genetic environment of *tet(X4)* in plasmid from *S*. 1,4,[5],12:i:- pJH3-*tet(X4)*. Comparative alignment of the genomic context surrounding *tet(X4)* on plasmid pJH3-*tet(X4)* with reference plasmids pYUSQPS29-1 (CP189877.1) and pJS-19S230 (CP130474.1). The upstream region of *tet(X4)* retains the canonical IS1R–RdmC–*tet(X4)* module observed in reference plasmids. In contrast, pJH3-*tet(X4)* lacks the downstream *expZ–catB3–*ISVsa3 cassette, instead continuing directly with a *ydhC–hdfR*–Tn2–IS26–*bla*_TEM − 1B_-*aadA22* module. Multiple insertion sequences (IS1R, IS15, IS26) are interspersed within this region, suggesting that IS26-mediated recombination either excised the cassette or prevented its integration. This remodeling resulted in a streamlined multidrug resistance island while maintaining core resistance determinants.

ZJJH22SAL03 harbors *tet(X4)* on a 187,186-bp multi-replicon plasmid [pJH3-tet(X4)] assigned to IncHI1/FIA (HI1)/HI1B (R27). The upstream region of *tet(X4)* retains the canonical IS1R–RdmC–*tet(X4)* configuration. In contrast to reference plasmids pYUSQPS29-1 (CP189877.1) and pJS-19S230 (CP130474.1), pJH3-tet(X4) lacks the intervening *expZ–catB3*–ISVsa3 cassette downstream of *tet(X4)*, and the downstream segment directly continues with the *ydhC–hdfR*–Tn2–IS26–*bla*_TEM − 1B_-*aadA22* module, interspersed with multiple insertion sequences (IS1R, IS15, IS26). This arrangement suggests that IS26-mediated recombination likely excised the canonical cassette or prevented its integration, resulting in a streamlined resistance region while maintaining core MDR determinants (Figure 2, Table 2).

**Table 2:** Genetic characterization of S. 1,4,[5],12:i:- strain ZJJH22SAL03.

**Replicon**	**Size (bp)**	**GC content (%)**	**Antimicrobial resistance genes**	**Plasmid replicon type(s)**	**GenBank accession**
Chromosome	4,978,368	52.2	*aph(3”)-Ib aph(6)-Id aac(6')-Iaa sul2 tet(B)*	—	CM129935.1
pJH3-*tet(X4)*	187,186	46.4	*aadA22 blaTEM-1B lnu(G) floR qnrS1 tet(X4)*	IncHI1/FIA (HI1)/HI1B (R27)	JBRUUC010000001.1
Plasmid2	3,373	55.2	—	—	JBRUUC010000002.1

**Figure 3:**
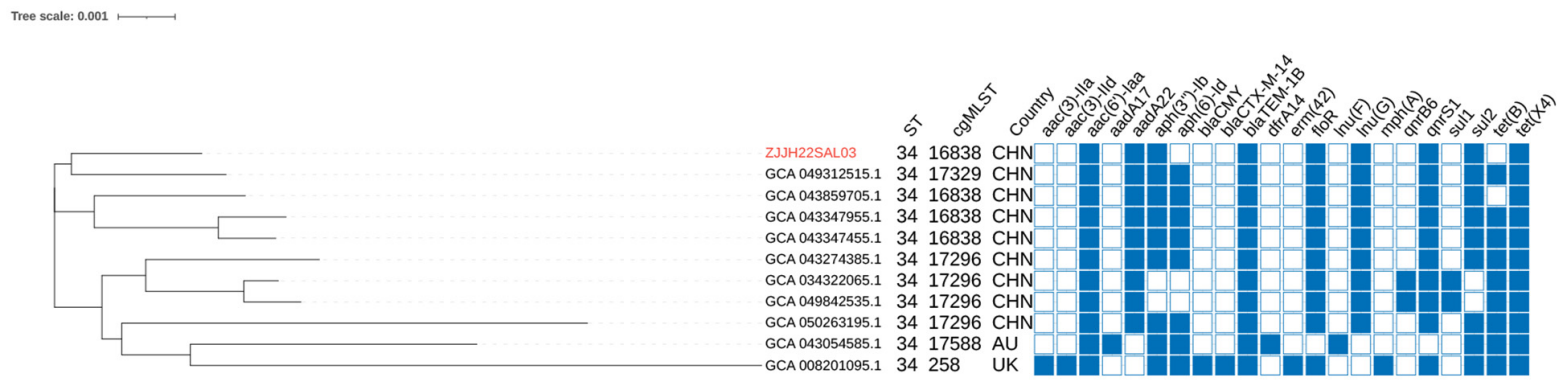
Phylogenetic structure and distribution of antimicrobial resistance gene categories among S.1,4,[5],12:i:- isolates from Jinhua, China. A NJ tree was constructed based on core-genome SNPs identified from 51 *S*. 1,4,[5],12:i:- isolates. The topology illustrates that all isolates belong to the ST34 lineage but cluster into multiple core genome sequence types (cgSTs), reflecting substantial genomic diversity within the regional population. Metadata rings adjacent to the tree denote sequence type (ST), cgST, sampling year, and city of origin. The outermost ring indicates the presence or absence of antimicrobial resistance genes grouped by major classes (aminoglycosides, β-lactams, sulfonamides, tetracyclines, phenicols, and others).

Phylogenetic analysis placed ZJJH22SAL03 within ST34/cgST-16838, forming a tight cluster with GCA_049312515.1 and related Chinese isolates, while a distinct cgST-17296 block and a divergent UK genome (branch length ~0.010) confirmed at least two independent introductions of *tet(X4)* into ST34 (Figure 3).

Conjugation assays demonstrated that pJH3-tet(X4) is self-transmissible to E. coli J53 at 3.33 × 10^−5^ transconjugants per donor. Transconjugants displayed tigecycline MICs >8 mg/L, representing a ≥16–64-fold increase compared with parental J53 (0.125–0.5 mg/L), thereby surpassing clinical breakpoints and abolishing tigecycline efficacy.

Comparative resistance gene analysis confirmed a conserved ST34 backbone (*aac(6')-Iaa, aph(3”-Ib/aph(6)-Id*, *bla*_TEM − 1B_, *sul2, tet(B), floR, qnrS1*), with *tet(X4)* variably integrated. ZJJH22SAL03 is distinguished by the co-localization of *tet(X4), lnu(G), qnrS1*, and *floR* within pJH3-tet(X4), yielding an expanded resistance repertoire.

### 3.5 ZJJH23SAL27 carrying *bla*_**NDM-5**_–high-resolution comparative genomics and phylogeny

ZJJH23SAL27 harbors *bla*_NDM−5_ on a 277-kb multi-replicon plasmid (pJH27-NDM) assigned to IncHI2/IncHI2A. The plasmid carries an extensive accessory resistome, including *bla*_NDM−5_, *bla*_OXA − 10_, *bla*_TEM − 1B_, multiple aminoglycoside-modifying enzymes [*aac(3)-IV, aph(4)-Ia, aadA1, aadA22, aadA2b*], *lnu(F), floR, qnrS1, cmlA1, ARR-2, tet(A), sul3*, and *dfrA14*, while the chromosome encodes only a limited baseline resistome [$aph(6)-Id,aph(3^{\prime \prime }-Ib,aac(6^{\prime })-Iaa,bla_{\text{TEM-1B}}$, *sul2, tet(B)*].

**Figure 4:**
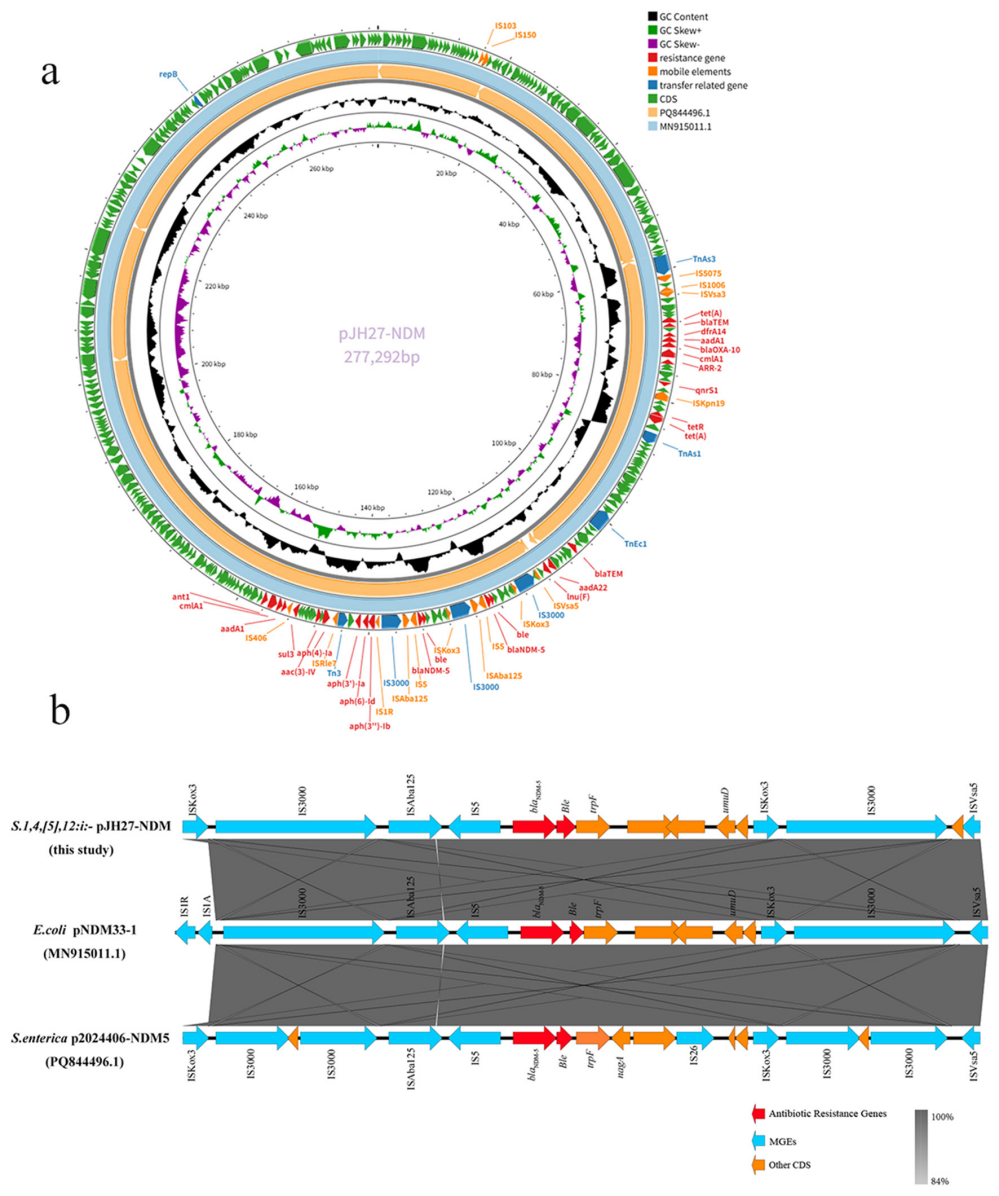
**(a)** Circular map of the *bla*_NDM−5_-bearing plasmid pJH27-NDM from *S*. 1,4,[5],12:i:- isolate ZJJD22SAL27. Circular representation of pJH27-NDM (277 kb, IncHI2/IncHI2A), showing the multidrug resistance gene repertoire carried on the plasmid. Resistance determinants include *bla*_NDM−5_, *bla*_OXA − 10_, *bla*_TEM − 1B_, aminoglycoside-modifying enzymes (*aac(3)-IV, aph(4)-Ia, aadA1, aadA22, aadA2b*), *lnu(F), floR, qnrS1, cmlA1, ARR-2, tet(A), sul3*, and *dfrA14*. **(b)** Genetic environment of *bla*_NDM−5_ in plasmid from *S*. 1,4,[5],12:i:- pJH27-NDM. Comparative alignment of the genomic context surrounding *bla*_NDM−5_ in pJH27-NDM with reference plasmids p2024406-NDM5 (CPQ844496.1) and pNDM33-1 (MN915011.1). In pJH27-NDM, the resistance island is structured as ISKox3–IS3000–ISAba125–IS5–*bla*_NDM−5_-*ble–trpF–umuD*, followed by ISVsa5. This arrangement largely resembles MN915011.1 but with ISKox3 replacing IS1R/IS1A, suggesting independent IS-mediated assembly. Structural variations in CPQ844496.1, including insertions (*nagA*, IS26) and truncated IS3000, highlight IS26/IS3000-driven remodeling of *bla*_NDM−5_ regions across hosts.

Comparative genomic analysis revealed that pJH27-NDM shares >99.9% nucleotide identity with reference plasmids p2024406-NDM5 (S. enterica, CPQ844496.1) and pNDM33-1 (E. coli, MN915011.1), but exhibits modular rearrangements within the *bla*_NDM−5_ locus. In pJH27-NDM, the resistance region is structured as ISKox3–IS3000–ISAba125–IS5–*bla*_NDM−5_-*ble–trpF*–umuD, flanked by ISKox3 and IS3000 and followed by ISVsa5. This configuration largely mirrors the canonical scaffold in MN915011.1, with ISKox3 replacing IS1R/IS1A, indicating independent IS-mediated assembly. Variations in CPQ844496.1, including additional insertions (nagA, IS26) and truncated IS3000 fragments, suggest IS26/IS3000-mediated structural diversification of *bla*_NDM−5_-bearing regions (Figure 4).

**Figure 5:**
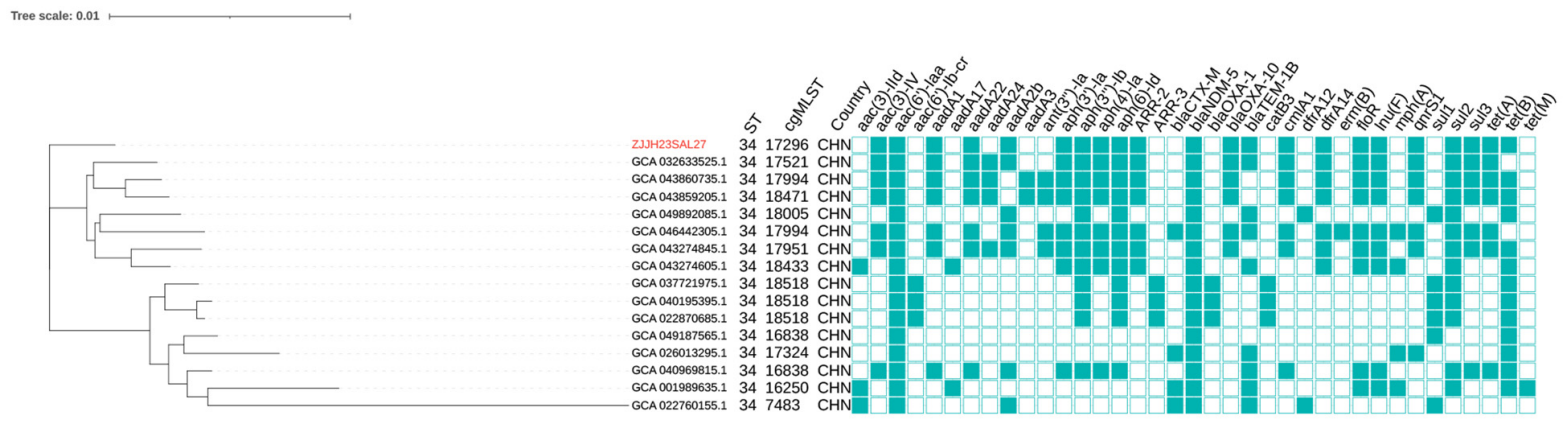
Phylogenetic analysis of *bla*_NDM−5_-positive *S*. 1,4,[5],12:i:- isolates from NCBI and ZJJH23SAL27. Core-genome phylogeny of *S*. 1,4,[5],12:i:- isolates carrying *bla*_NDM−5_. ZJJD22SAL27 (cgST-17296) forms a distinct single-tip branch closely related to other Chinese ST34 isolates. *bla*_NDM−5_ was identified across multiple cgSTs (e.g., 17296, 16838, 17521, 17951, 17994, 18005, 18433, 18471, 18518, 16250, 7483), indicating polyphyletic acquisition rather than clonal expansion. Resistome profiling confirmed co-occurrence of *bla*_NDM−5_ with *qnrS1, floR, sul3, tet(A)*, and aminoglycoside resistance genes, highlighting multiple co-selection pathways that facilitate persistence and spread under diverse antimicrobial pressures.

Phylogenetic analysis placed ZJJH23SAL27 within ST34/cgST-17296, forming a distinct single-tip branch adjacent to other Chinese ST34 isolates. *bla*_NDM−5_ was detected across multiple cgSTs (e.g., 17296, 16838, 17521, 17951, 17994, 18005, 18433, 18471, 18518, 16250, 7483) (Figure 5, Table 3).

**Table 3:** Genetic characterization of S. 1,4,[5],12:i:- strain ZJJH22SAL27.

**Replicon**	**Size (bp)**	**GC content (%)**	**Antimicrobial resistance genes**	**Plasmid replicon type(s)**	**GenBank accession**
Chromosome	5,023,947	52.2	$aph\left(6\right)-Idaph\left(3^{\prime \prime }\right)-Ibaac\left(6^{\prime }\right)-Iaabla_{\text{TEM}-1\text{B}}$ *sul2 tet(B)*	—	CM127137.1
pJH3-tet(X4)	277,292	47.3	$aph\left(4\right)-Iaaph\left(6\right)-Idaph\left(3^{\prime }\right)-Iaaph\left(3^{\prime \prime }\right)-IbaadA22aadA1aadA2baac\left(3\right)-IVbla_{\text{TEM}-1\text{B}}$ *bla*_NDM−5_ *bla*_OXA − 10_ *lnu(F) cmlA1 floR qnrS1 ARR-2 tet(A) sul3 dfrA14*	IncHI2/IncHI2A	JBQWED010000001.1
Plasmid2	1,888	51.3	—	—	JBQWED010000002.1

Conjugation assays demonstrated that pJH27-NDM is highly transmissible. At 37 °C, transfer occurred at 3.3 × 10^−3^ transconjugants per recipient, while at 26 °C—conditions favorable for IncHI2 plasmid transfer—the frequency increased to 0.2, representing a >60-fold enhancement.

### 3.6 Prophage and accessory dissemination determinants

To explore factors potentially contributing to the dissemination success of ST34 *S*. 1,4,[5],12:i:-, we analyzed prophage content, heavy metal resistance genes, *sopE*, and class 1 integrons across all 51 isolates.

Arsenic and mercury resistance determinants were detected in all 51 isolates (100%). Copper (*pco*) and silver (*sil*) operons were present in 49 isolates (96.1%) and showed complete linkage. Two isolates (ZJJH23SAL17 and ZJJH24SAL39) lacked both operons. The *sopE* virulence gene was detected in 33 isolates (64.7%). Class 1 integrons, defined by the presence of the intI1 integrase gene, were identified in 23 isolates (45.1%).

## 4 Discussion

*S*. 1,4,[5],12:i:- has emerged as one of the most successful clonal groups in recent decades, disseminating globally through foodborne transmission chains. Its epidemiological success is largely attributable to the adaptive flexibility of the ST34 backbone, which promotes the acquisition and stable maintenance of multidrug resistance (MDR) determinants via mobile genetic elements (Huang et al., 2025). In this study, we investigated 51 clinical isolates collected from diarrheal patients across Jinhua, Zhejiang Province, China (2022–2024), providing a regional genomic snapshot of ST34 circulation and diversification in eastern China.

From an epidemiological perspective, the isolates showed both demographic and temporal clustering. The majority of infections occurred in children under 3 years (39.2%) and elderly individuals (31.4%), reflecting the heightened susceptibility of immunologically immature and senescent populations. A marked summer peak (July–September) coincided with elevated ambient temperatures and increased foodborne exposure risk, suggesting that environmental factors may amplify seasonal transmission. Geographically, cases were distributed across seven counties, with Yongkang identified as a local hotspot, indicating possible shared exposure routes or community-level amplification.

Antimicrobial susceptibility testing revealed persistently high resistance to long-used agents such as ampicillin, tetracycline, chloramphenicol, and trimethoprim-sulfamethoxazole, consistent with the canonical MDR profile of ST34 (Petrovska et al., 2016; Seixas et al., 2016). This phenotype correlated with a conserved resistance gene set (*bla*_TEM − 1B_, *sul2, tet(B), floR, aac(6')-Iaa*) that was ubiquitous among isolates. In contrast, resistance to third-generation cephalosporins, fluoroquinolones, carbapenems, and azithromycin remained sporadic (< 20%), suggesting that selective pressure from legacy antimicrobials continues to dominate the resistome landscape. All isolates were susceptible to polymyxin B, underscoring its retained clinical efficacy. A gradual decline in resistance to cefotaxime and chloramphenicol from 2022 to 2024 was observed; however, the underlying drivers remain uncertain and may involve changes in antimicrobial usage or other epidemiological factors.

Despite this stability, genomic analyses demonstrated progressive diversification of both phylogeny and resistome composition. Early isolates from 2022 were mostly confined to cgST-16838 and carried a limited gene set, whereas isolates collected in 2023–2024 displayed greater phylogenetic heterogeneity (cgST-17296, 17521, 18410, 9106) and an expanded accessory resistome that included *bla*_CTX − M−55_, *fosA3, erm, ARR* genes, and notably, the last-line resistance determinants *tet(X4)* and *bla*_NDM−5_. The detection of these two genes within the same regional population is noteworthy and may indicate the presence of high-risk resistance determinants with potential clinical relevance.

The isolate ZJJH22SAL03 carried *tet(X4)*, which encodes a flavin-dependent monooxygenase capable of enzymatic inactivation of tigecycline (Xu et al., 2019), leading to a ≥32-fold increase in MICs (MIC >8 mg/L) and effectively abolishing tigecycline efficacy. Conjugation assays further demonstrated that the *tet(X4)*-carrying plasmid was self-transmissible (3.33 × 10^−5^), confirming its potential for horizontal dissemination. The co-localization of *floR* and *qnrS1* on the same plasmid suggests that non-tigecycline selective pressures, such as florfenicol or fluoroquinolone use, may facilitate the persistence and spread of *tet(X4)* across agricultural and clinical settings (He et al., 2019; Yang et al., 2023). Plasmid-mediated *tet(X4)* was first identified in a *Salmonella enterica* isolate from China in 2016 (Wang et al., 2021) and has since been reported in both *Salmonella* and *Escherichia coli* from human and animal sources (Yan et al., 2024), highlighting the potential for cross-species dissemination.

From a structural perspective, the plasmid architecture of *tet(X4)* in ZJJH22SAL03 [pJH3-tet(X4)] differs from several previously described contexts. For example, in a *Salmonella enterica* serovar Llandoff plasmid, *tet(X4)* was flanked by two ISCR2 elements, forming a putative mobile unit that may enhance interspecies transfer (Wang et al., 2021). In contrast, pJH3-tet(X4) lacks this canonical ISCR2-bracketed cassette and instead contains multiple insertion sequences (IS1R, IS15, IS26) surrounding *tet(X4)*, together with a streamlined resistance region. Similar diversity has been observed in Enterobacteriaceae, where *tet(X4)* may occur in arrangements such as IS26–abh–*tet(X4)*–ISVsa3 and is often linked to additional resistance genes including *floR* and *qnrS1*.

These observations highlight the structural plasticity of *tet(X4)*-bearing plasmids and suggest that distinct IS-mediated recombination events continue to reshape their genetic environments. Such flexibility likely facilitates the ongoing adaptation and dissemination of tigecycline resistance within the ST34 lineage.

Equally concerning was the detection of *bla*_NDM−5_ on an IncHI2 plasmid in isolate ZJJH23SAL27. IncHI-type plasmids (HI1 and HI2) are known to be temperature-sensitive, with optimal transfer at 25–30 °C but markedly reduced conjugation at 37 °C. Consistent with prior studies (e.g., plasmids pSTM6-275 and R27) (Billman-Jacobe et al., 2018; Gibert et al., 2020; Taylor, 2009), our conjugation assays demonstrated a >60-fold increase in transfer efficiency at 26 °C (0.2 vs. 3.3 × 10^−3^ at 37 °C), suggesting that environmental and food-related temperatures could promote horizontal dissemination of carbapenemase genes. This finding is consistent with the possibility that food-handling and processing environments could contribute to plasmid dissemination, although direct evidence was not assessed in this study.

Phylogenetic placement of ZJJH23SAL27 as a distinct branch within ST34 supports independent plasmid acquisition events rather than clonal expansion. The *bla*_NDM−5_ plasmid also carried multiple accessory determinants (*qnrS1, floR, sul3*, aminoglycoside-modifying enzymes) within IS26-mediated modular structures, a configuration that enhances plasmid stability and adaptability even in the absence of carbapenem exposure (Lv et al., 2022). The convergence of *bla*_NDM−5_ acquisition within the globally dominant ST34 lineage therefore represents a concerning but plausible evolutionary trajectory, and warrants continued surveillance (Deng et al., 2024; Tan et al., 2024).

In addition to antimicrobial resistance, prophage-associated and accessory fitness determinants may also contribute to the ecological success of ST34 (Tassinari et al., 2020). In particular, acquisition of the sopE gene via lysogenic phages has been proposed to enhance host colonization, macrophage survival, and overall ecological fitness, and has also been linked to the monophasic phenotype and associated genomic remodeling (Charity et al., 2022; Ingle et al., 2021).

Heavy metal resistance determinants were highly prevalent in our collection, with arsenic and mercury resistance genes present in all isolates and the pco–sil operons detected in the vast majority. Because heavy metal tolerance has been associated with co-selection under agricultural and environmental metal exposure, their widespread distribution is consistent with the possibility that non-antibiotic environmental pressures may contribute to the maintenance of ST34 clones (Vázquez et al., 2023; García et al., 2016). However, as environmental metadata were not available, this interpretation should be made with caution.

Class 1 integrons were identified in 45.1% of isolates based on intI1 carriage. Previous studies suggest that class 1 integrons, often associated with relatively weak promoters, may provide a fitness advantage by enabling balanced expression of resistance gene cassettes (Okoh and Fadare, 2022; Garrido et al., 2024). The moderate prevalence observed here indicates that integrons may contribute to multidrug resistance in a subset of ST34 isolates but are not universal features of this lineage in our setting.

Taken together, these observations suggest that prophage-associated virulence factors, heavy metal resistance, and integrons may collectively shape the adaptive landscape of ST34 *Salmonella*. Nonetheless, their variable distribution among isolates indicates that the success of this lineage is likely multifactorial and context-dependent rather than driven by any single determinant.

Collectively, these findings suggest the presence of dual evolutionary patterns within the *S*. 1,4,[5],12:i:- ST34 population in Jinhua: (i) maintenance of a stable MDR backbone conferring resistance to common antimicrobials, and (ii) episodic acquisition of high-risk plasmids carrying *tet(X4)* and *bla*_NDM−5_. This pattern is consistent with the ecological flexibility of ST34 as a lineage capable of maintaining established resistance traits while intermittently incorporating new ones. The predominance of infections among children and the elderly, together with the observed plasmid transfer under food-relevant temperatures, highlights the potential public health relevance of continued genomic surveillance. Co-detection of *tet(X4)* and *bla*_NDM−5_ within the same regional population warrants attention, although their current frequency remains low. Continued monitoring is therefore important to assess whether these determinants become more widespread over time.

This study has several limitations. First, isolates were collected through a sentinel hospital network and may not fully represent the broader population or capture community-wide transmission dynamics. Second, the annual sample size was modest (*n* = 11–23), which limits statistical power for detecting temporal trends. Third, resistance gene identification relied on curated databases that may not capture novel or uncommon determinants. Fourth, the geographic origins and transmission routes of plasmids and strains cannot be resolved from genomic data alone, and potential contributions from travel, food importation, or interregional transmission were not evaluated. Finally, the observational design precludes causal inference regarding selective pressures. These considerations should be taken into account when interpreting the findings.

## 5 Conclusions

This study demonstrates the persistence of a multidrug-resistant core and the occasional occurrence of last-line resistance genes *tet(X4)* and *bla*_NDM−5_ among *S*. 1,4,[5],12:i:- ST34 isolates in eastern China. The identification of a self-transmissible *tet(X4)*-bearing plasmid and a temperature-sensitive *bla*_NDM−5_-carrying IncHI2 plasmid indicates the capacity of this lineage to acquire and maintain clinically relevant resistance determinants. These observations support the value of continued genomic surveillance under a One Health framework to monitor plasmid dynamics and the distribution of high-priority resistance genes in *Salmonella* populations.

## Data Availability

The datasets presented in this study can be found in online repositories. The names of the repository/repositories and accession number(s) can be found below: https://www.ncbi.nlm.nih.gov/genbank/, PRJNA1311943 https://www.ncbi.nlm.nih.gov/genbank/, PRJNA1312021 https://www.ncbi.nlm.nih.gov/genbank/, PRJNA1312031.
